# Winter pressures in general practice in England

**DOI:** 10.3399/BJGP.2025.0799

**Published:** 2026-02-01

**Authors:** Rachel E Denholm, Luisa M Pettigrew, Shrinkhala Dawadi, Emily Herrett, Ruth E Costello, Mengxuan Zou, Rosalind M Eggo

**Affiliations:** 1 Bristol Medical School, Population Health Sciences, University of Bristol, Bristol, UK; 2 Health Data Research UK South West, Bristol, UK; 3 NIHR Bristol Biomedical Research Centre, Bristol, UK; 4 Faculty of Epidemiology and Population Health, London School of Hygiene & Tropical Medicine, London, UK; 5 The Health Foundation,, London, UK

Every winter, the NHS braces for surges in demand, with effects across the system. However, focus has often centred on hospitals, but the reality is that much of this seasonal strain often first lands in general practice, when practices are already facing growing demand for consultations, greater clinical complexity, and significant administrative burdens.^1^ Despite its central role, winter pressures in general practice remain poorly characterised, as do the consequences of pressure on individual practices, on patient health, and on the wider system.^2^ GPs already regularly report working at or beyond capacity, hence there is little flexibility to manage seasonal spikes.^1^ Preliminary results from our NIHR-funded study, which has surveyed general practices in England (*n* = 191), shows rising pressure leading up to winter: 30% of general practices (*n* = 28/94) reported being very busy to burnt out in mid-October, increasing to almost 50% (*n* = 94/191) by early December (Dawadi, *et al*, unpublished data, 2025).^3^ Trends vary by practice, likely reflecting differences in patient case-mix, local respiratory epidemics, and how practices and demand are resourced and organised. Without national-level understanding and quantification of practice level pressures and their effect on the wider health ecosystem we cannot plan effectively, allocate resources fairly, or protect patient care during the most challenging months of the year.

## Why it matters now

National policy seeks to shift activity into the community,^4^ yet practices are already managing an ageing population, rising multimorbidity, increasing clinical complexity, and workforce shortages.^5^ When patients cannot access general practice, they turn to urgent and emergency services, further adding to demand on an already congested system, or give up trying to access care, leading to worse outcomes.^6^ Recent attempts to use operational pressure tools in primary care, such as Operational Pressure Escalation Levels (OPEL), as used in hospitals, have offered limited practical benefit, with GPs reporting that these systems neither relieve workload nor prompt meaningful support.^7^ If winter pressures are to be addressed rather than endured, general practice needs a shift from reactive crisis response to proactive, evidence-informed planning that reflects its central role in maintaining the stability of the NHS.

## Gaps in understanding of winter pressure

Despite its importance, we have no reliable way to measure winter pressures at practice level, nor to understand how practices respond to them, how they affect patients or shape demand across the wider health and care system.

Appointment numbers capture recorded consultations in general practice, and show seasonal peaks in October, around flu vaccination, and in March, ahead of Quality and Outcomes Framework deadlines, indicating increases in supply are likely driven by financial incentives.^8^ However, appointment figures provide only a partial view of workload. They overlook complexity of care provided, the substantial volume of undocumented activity that occurs in general practice, and miss unmet demand, such as unanswered calls, abandoned online requests or patients who bypass their GP altogether, delay seeking care or do not seek care because access feels too difficult.

Workforce data^9^ allows us to calculate patient-to-staff ratios, but vacancy rates in general practice are not reported, making it impossible to distinguish between recruitment shortages and deliberate staffing decisions. While the Care Quality Commission assesses the ‘responsiveness‘ of practices’ ,^10^ this does not necessarily examine a practice’s capability to manage surges in demand, for example during winter pressures. Consequently, we have limited insight into how practices organise themselves to respond to fluctuating demand under an already strained system.

Although reduced access to general practice has been associated with higher emergency department attendance,^6^ we still don't know how pressure within general practice affects patients and the wider system. Continuity and relational care, known to improve outcomes and reduce mortality, are often sacrificed to prioritise speed and volume of access. Nor do we understand how short-term workload fluctuations in general practice influence hospital capacity, delayed discharge, or community and social services. Winter pressures are unlikely to fall evenly: deprivation, multimorbidity, and limited social support increase vulnerability, yet we lack the granular and joined-up data needed to identify who is most affected and how inequalities widen. Without addressing these gaps, winter planning will remain reactive and unable to deliver the resilience the system needs.

## Opportunities

New opportunities to understand winter pressure in general practice are beginning to emerge. Routine appointment data now offer greater visibility of activity, and patient-level electronic health record data can provide insights into the reason for presentation, likely complexity, and outcomes — specially when records are linked across the health system. Newly available indicators from cloud-based telephony^11^ and online consultation submissions^12^ can generate real-time information on demand, particularly as practices are now required to keep online routes open throughout core hours. However, these data are currently designated as ‘statistics in development’ and interpreting them remains challenging, they also risk overlooking unmet need among groups already disadvantaged by digital access. Variation between practices also warrants attention, as these indicate organisations implementing different mitigating measures, and understanding the contribution of workforce mix, organisational processes, and technology could inform more effective support. The expansion of digital tools, and opportunities to link to patient-level health records, must also offer opportunities to measure winter pressures better.

## A call to action

Winter pressures are predictable. Treating them as an annual crisis is not sustainable. Better intelligence is necessary, but not sufficient: data must underpin coordinated strategies that anticipate pressure, evaluate management strategies, and target support, recognising that capacity is finite and that sustainable solutions depend on alignment across the health and care system. Our NIHR-funded study on inequalities in winter pressures in general practice is a step toward this goal, but research alone is not enough.^3^ Policymakers must commit to building the data infrastructure and operational capacity that modern general practice requires.
